# Interfacing External Quantum Devices to a Universal Quantum Computer

**DOI:** 10.1371/journal.pone.0029417

**Published:** 2011-12-28

**Authors:** Antonio A. Lagana, Max A. Lohe, Lorenz von Smekal

**Affiliations:** 1 School of Chemistry and Physics, University of Adelaide, Adelaide, South Australia, Australia; 2 School of Chemistry and Physics, University of Adelaide, Adelaide, South Australia, Australia; 3 Institut für Kernphysik, Technische Universität Darmstadt, Darmstadt, Germany; University of Nottingham, United Kingdom

## Abstract

We present a scheme to use external quantum devices using the universal quantum computer previously constructed. We thereby show how the universal quantum computer can utilize networked quantum information resources to carry out local computations. Such information may come from specialized quantum devices or even from remote universal quantum computers. We show how to accomplish this by devising universal quantum computer programs that implement well known oracle based quantum algorithms, namely the Deutsch, Deutsch-Jozsa, and the Grover algorithms using external black-box quantum oracle devices. In the process, we demonstrate a method to map existing quantum algorithms onto the universal quantum computer.

## Introduction

Quantum networks which connect quantum systems and can transmit quantum information have been extensively discussed [1]. Quantum connectivity provides a means of overcoming size-scaling and error-correction problems, and has significant advantages over classical connectivity. Furthermore, networks of quantum computers have also been proposed [2] where information can be exchanged between nodes via quantum and classical channels. A general question arises as to whether and how such quantum computers can communicate and exchange information. In the simplest case a quantum computer may download data sets from other nodes over the quantum network, but in more complex cases use the network to call subroutines, or concatenate programs from other quantum computers.

It is well known that classical principles do not necessarily apply in the realm of quantum mechanics. The no-cloning theorem (see [3] for example) is a well-known example of this. In the field of quantum computing, the ability to halt a programmable quantum computer was such an example. The original Universal Quantum Turing Machine proposal [4] made the tacit assumption that a quantum turing machine could be halted in a classical manner. This turned out to be problematic (see [5] for a discussion of the issues associated with the original proposal) due to properties of quantum mechanics. Thus, it is imperative to formally show whether a classical solution or property is applicable (or even relevant) in the realm of quantum mechanics. Assuming that a classical solution to a problem directly applies to a quantum mechanical system is prone to run into potential complications.

We address here the question of how a universal quantum computer can access an external oracle, which may be regarded as a “black box” quantum device, possibly over a quantum network but in any case as a separate and external quantum system to the universal quantum computer itself. In fact, the oracle may be a program running on a remote universal quantum computer. It should be noted that this is a different problem from that of implementing an oracle “program” on a universal quantum computer. This is of course possible by virtue of the fact that the computer is universal. Hence, if such a program exists, it can be implemented and executed on a universal quantum computer. Strictly speaking, however, the ability to utilize external quantum devices over a network connection is a different problem because such devices are external to the universal quantum computer itself.

Classically, the ability to access devices on a network is a well-known problem with well-known solutions. However, as stated earlier, we cannot assume that this is necessarily the case for a quantum computer accessing quantum devices on a quantum network. Our aim is to explicitly show that accessing external quantum devices with a universal quantum computer is indeed possible by devising universal quantum computer programs that implement well-known oracle based quantum algorithms, namely the Deutsch, Deutsch-Jozsa, and the Grover algorithms using external black-box quantum oracle devices.

In [5] we constructed a programmable universal quantum computer 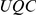 that is universal in the sense that it can emulate any classical Turing machine and can approximate any unitary operation to any desired accuracy. It is programmable in the sense that the machine's operations are specified using a sequence of instructions in the same way as for classical computers. 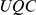 also supports conditional branching and hence conditional execution, a feature that is not directly possible in the quantum gate array circuit framework. Moreover, 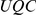 uses a halting scheme that allows for valid program concatenation, thus resolving issues with the original Universal Quantum Turing Machine (UQTM) proposed by Deutsch [4].

In order to use information from a quantum network in 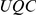 programs, we need to devise a means of enabling 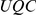 programs to access such remote information and use that information for local computations. We assume that remote quantum nodes exist and treat them as black boxes without any assumptions as to their internal structure or operational details. Without loss of generality, we assume that such devices accept a finite number of input qubits and generate a finite number of output qubits. The input and output qubits may be shared, which is the case if the remote device functions in such a way as to alter the input qubits based on its function. We also assume, without loss of generality, that quantum network nodes have an “enable” qubit, 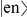, that controls when an access is to begin, in order to let the device know when the input data has been prepared and is valid. We further assume, without loss of generality, that the nodes of the network generate their output data in less time than the time associated with a single iteration of 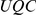. If the query time were longer than a single iteration of 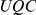 or were data-dependent, one could simply write the 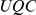 program to wait for the appropriate number of cycles before using the result of the network access. Alternatively, the nodes could provide an “access completed” status flag qubit such that the 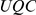 program could poll this status flag qubit before using the result of a network access.

## Results

Recall from [5] that 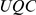 consists of a memory tape 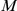 with an infinite number of qubits, of which only a finite portion is ever used, and a processor that contains observables that play the roles of several registers, including a data register 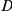, a program counter register 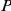, a scratch qubit 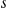, and the halt qubit 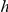. The processor executes programs stored on the memory tape using data that is also stored on the memory tape. A program of 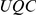 consists of a sequence of qubits whose states encode instructions of the instruction set defined in [5] and reproduced in Table 1 at the end of this paper.

**Table 1:** 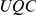
 Instruction Set.

Label	Encoding	Description
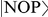	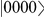	No operation
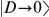	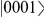	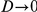
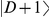	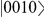	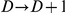
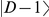	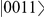	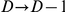
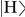	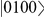	Apply Hadamard operation to 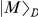
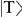	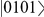	Apply 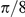 operation to 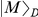
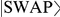	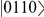	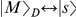
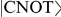	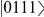	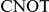 of 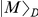 and 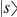 ( 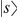 : control)
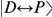	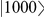	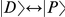 (branch) iff 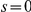
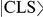	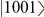	Clear 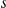
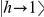	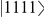	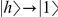 (set halt qubit)

The single qubit operations 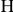 and 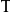 act on the qubit at tape location 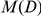, denoted 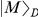, and the two qubit operations 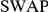 and 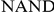 act on 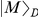 and the scratch qubit 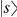, the latter being used as the control qubit for the 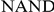 operation.

The instruction set includes a set of operations that can approximate any unitary operation to any desired accuracy. Thus, it is quantum computationally universal. In [5] we constructed a 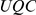 program that can compute the 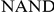 function, thereby showing that the machine can compute any classically computable function. Because of 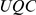
's universality, any algorithm that can be implemented in the quantum gate array framework can be mapped to an equivalent 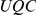 program by virtue of the fact that gate array circuits can be decomposed into circuits of gates with the same universal set of unitary operations 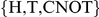 that are implemented in the 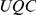 instruction set. Each of the qubits in a quantum circuit (i.e. lines connecting gates) can be mapped to a suitable memory tape data qubit and each of the unitary operations (i.e. quantum gates) can be mapped to a suitable 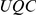 subroutine. It is possible therefore to map quantum gate array implementations of algorithms such as the quantum Fourier transform, quantum phase estimation, quantum order finding, quantum factoring discussed in [6] (Chapter 5) onto 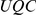.

### Accessing Networked Quantum Resources With 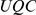


Modifying 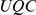 to use networked quantum devices, then, is a matter of connecting the qubits comprising the interface (input, output, enable, and optionally access complete) qubits of those devices to a finite subset of the data portion of 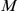, which is the quantum analog of a classical computer's memory-mapped I/O and allows 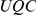 programs to access remote devices using the 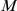 qubits that are connected to those devices. The 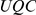 programs prepare the appropriate input data qubits, set the corresponding access enable qubits to perform an access, and utilize the corresponding output data qubits of 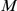. It should be noted that a remote quantum device could be another instance of 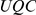 which would enable distributed quantum computing. However, the scheme to access data from remote devices, be they simple devices or full-fledged quantum computers, would work in the same way.

### Primitive Programs

In [5] we defined several primitive programs and subroutines that serve as building blocks for devising and analyzing more complicated and useful programs. We reproduce here only those that we specifically require for constructing the algorithms that are the focus of this work. By considering the quantum gate array framework implementations of the algorithms, we identify that we need programs that perform the operations 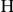, 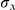, and 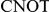. We also need to swap qubits for several operations such as enabling or disabling the remote networked quantum device, and the ability to address individual qubits on the memory tape to perform operations on them. Finally, we need a primitive program to halt the overall program.

In the equations that follow, superscripts on programs denote the operation specified by the program and subscripts indicate the qubits on which the program specifies the processor to operate upon. For notational simplicity, 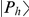 denotes the program that halts 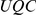, i.e. 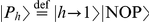.

The first set of primitive programs, 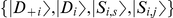, is a subset of those defined in [5]:

1. 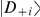: Increment 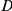 by 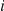,
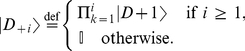
(1)


2. 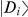: Set 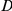 to 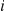, 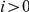,
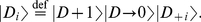
(2)


Recall from the discussion of 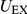 in [5], that we precede the 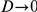 instruction with a 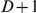 instruction to ensure that 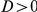 when the 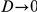 instruction is executed.

3. 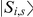: Swap data qubits 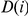 and 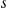,
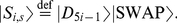
(3)


4. 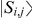: Swap data qubits 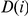 and 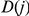,
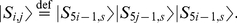
(4)


We also describe the set of programs 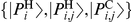 which apply the single- and multiple-qubit 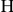 and 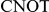 operations on arbitrary qubits on the memory tape, where 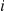 and 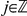:

1. 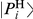: Apply 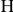 to data qubit 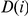,
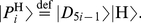
(5)


2. 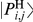: Apply 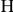 to data qubits 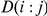, where 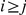,
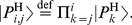
(6)


One could implement this program using a loop but that would require first implementing binary addition of 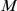 qubits. Binary addition is possible because one can implement a binary adder such as a Carry Lookahead Adder (CLA) [7] using the 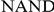 program that we defined in [5]. However, since we are only interested in a polynomial order (in the number of qubits) multiple qubit Hadamard transformation program, we define 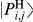 as a sequential “unrolled” loop program.

3. 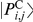: Apply 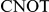 to data qubits 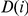 and 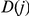 with 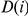 as the control qubit,
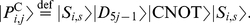
(7)


Using the primitive programs defined above, we define 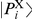 as the program that applies the 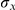 (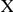) operation on data qubit 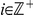. Noting that a 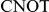 operation with the control qubit in the 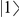 state is equivalent to the 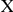 operation, we deduce the equivalence
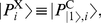
(8)where the subscript 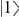 denotes that some suitable data qubit on the memory tape has been prepared in the state 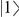. Similarly, we define 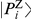 as the program that applies the 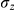 (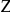) operation on data qubit 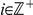. Noting that 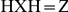, we deduce
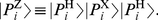
(9)


Finally, we define a program 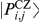 that conditionally applies the 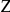 operation on data qubit 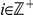 and data qubit 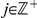. Since 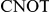 is the conditional 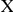 operation, we have
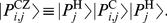
(10)


### 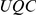 Algorithms Using Networked Quantum Oracle Devices

With the notable exception of Shor's factorization algorithm [8], several well known quantum algorithms that achieve a speed-up over their fastest known classical counterparts rely on the use of an oracle, the best known examples being the Deutsch, Deutsch-Jozsa, and Grover algorithms (see Nielsen and Chuang [6], for example). The Deutsch algorithm can determine a global property of a function 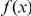, namely 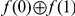, using only one evaluation of 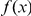 whereas the fastest classical algorithm requires at least two evaluations of 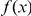. The Deutsch-Jozsa algorithm can determine whether a two-valued (0 or 1) function 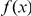 is constant or balanced with only one evaluation of 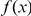 whereas the fastest classical algorithm requires 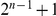 evaluations, where 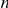 denotes the number of bits required to encode the possible values of 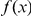. Grover's algorithm [9] can find a marked item in an unstructured database of 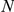 elements in 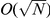 operations whereas the fastest classical algorithm requires 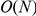 operations. Thus, these quantum algorithms all achieve at least a quadratic speedup over their classical counterparts.

These algorithms are well suited to illustrate the use of networked quantum resources with the 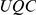 because they rely on black-box quantum devices that generate some output based on the given input. They thus serve as prototypical examples of a networked quantum node, whose internal implementation details are unknown; only the interface protocol need be known. Here, we assume the simplest protocol, which is that the output is valid one “clock cycle” after making a request.

#### Deutsch and Deutsch-Jozsa Algorithms on 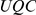


We now illustrate the use of a networked quantum device in a 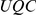 program by first implementing the simplest known oracle based quantum algorithm, Deutsch's algorithm. The Deutsch oracle works as follows:
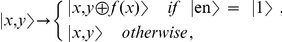
where 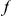 is some function and 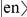 denotes the oracle query enable flag. The memory tape is prepared with 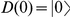 and 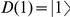 where 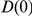 and 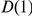 take the roles of 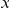 and 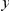, respectively. We assume without loss of generality that 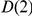 takes the role of 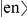 and is prepared as 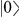, and 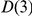 is initially prepared as 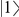.

The program that executes the Deutsch algorithm is
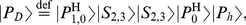
(11)where 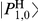 applies the Hadamard transform to the data qubits corresponding to 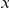 and 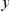. 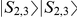 swap qubits 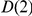 and 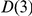 thereby setting the oracle's 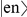 qubit (recall that 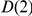 is connected to 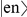 and that 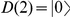 and 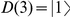 initially) for a single 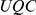 cycle and then clears it, returning the state of 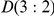 back to the original state. At this point, the oracle has generated the output state 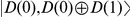. 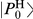 then applies the Hadamard transform to the 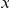 output of the oracle and 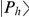 halts the program thus yielding the following on the memory tape:
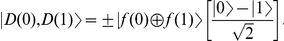



Measuring 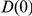 yields the result that we were interested in, 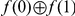. This is a specific mapping of the gate array implementation of the algorithm (see [6] Figure 1.19, for example) onto the instruction set of 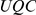.

We can similarly implement the Deutsch-Jozsa algorithm by mapping a gate array implementation such as the one shown in [6], Figure 1.20. In this case, data qubits 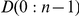 take the role of 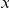, 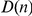 takes the role of 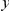, and we use 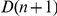 as the 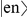 qubit. As before, 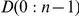 are prepared in the 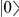 state, 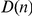 is prepared in the 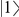 state, 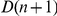 is prepared in the 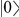 state and 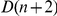 is prepared in the 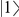 state. The Deutsch-Jozsa oracle works like the Deutsch oracle with the only difference being that 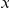 is 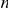 qubits wide. The resulting 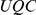 program that computes the Deutsch-Jozsa algorithm is therefore
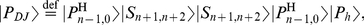
(12)


which is again a direct mapping of the gate array implementation onto the 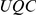 instruction set.

#### Grover's Algorithm on 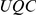


We now use the techniques developed in the previous section to implement the Grover unstructured database search algorithm. We assume that the database has only one marked solution as can be determined by using the quantum counting algorithm (see [6] Chapter 6, for example). We denote the query data qubits as 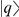 and the query enable flag as 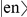. The Grover oracle works as follows:
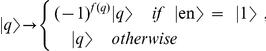
where 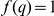 if 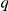 is a solution to the search problem and 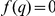 otherwise. More concisely, the oracle performs the unitary transformation
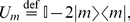
(13)where 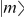 denotes the marked solution. In other words, the oracle flips the phase of the solution state but leaves non-solution states unchanged. Grover's algorithm prepares an initial query state as the equal superposition of all elements in the database, followed by 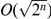 iterations of 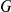, where
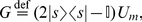
(14)


and
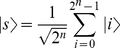
(15)


denotes the equal superposition of all database elements.

Thus, the first step in the program is to create a superposition of all database items in 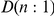 where 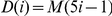, 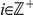, as the first query input. This is accomplished by the multiple qubit Hadamard primitive program 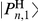 defined in Eq. (6). The next step is to perform an oracle query. The following program performs an oracle call with query data prepared in 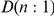:
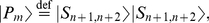
(16)where 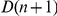 is used as the oracle query enable qubit and 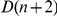 is initialized to 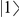. 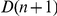 is assumed to be initialized to 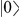 (i.e. the oracle query data is disabled at start-up). This program simply sets the query enable qubit for a single 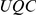 cycle and then clears it, returning the state of 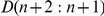 back to the original state. Thus, upon running 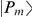, the result of the oracle call is in 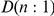, i.e. this program is functionally equivalent to 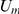.

The next step is to implement a program 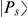 that performs the reflection of a given state about the superposition of all basis states 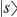. This requires a conditional-phase operation that works as follows:
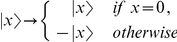
where 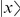 is 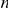 qubits wide. Up to a global phase, this can be implemented using the following procedure:

1. Apply the 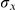 operation to all 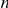 qubits.

2. Apply a controlled-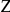 operation using 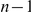 qubits as control qubits and the remaining qubit as the data qubit.

3. Apply the 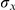 operation to all 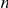 qubits.

We can construct a multiple qubit controlled-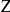 program 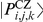 where qubits 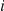 through 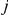 are the control qubits and qubit 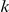 is the data qubit, with the 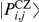 program defined in Eq. (10) and the Toffoli program 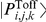 that we defined in [5] using a procedure analogous to that described in [6], Chapter 4. Armed with 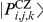, we construct 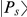 as follows:
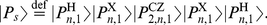
(17)


It can be readily verified that this is functionally equivalent to the 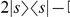 operator. Thus, a program that performs a single Grover iteration is
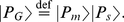
(18)


In summary, the complete program to search a database of 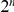 items with a single marked solution is
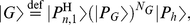
(19)where 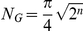 is the number of Grover iterations that can be pre-computed based on the database size, or that 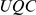 can compute from the database size using a classical algorithm. Upon execution of 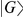, a measurement of 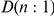 reveals the solution 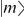. Because there are no oracle queries associated with 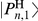 and 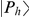, we immediately identify the complexity (as a measure of the number of oracle queries) of 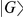 as 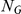. As is to be expected, this complexity is identical to the number of oracle queries associated with an implementation in the gate array framework.

## Discussion

We have presented a scheme to allow universal quantum computers to utilize networked quantum resources. We have illustrated the scheme by devising 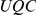 programs that implement the well-known oracle based Deutsch, Deutsch-Jozsa, and Grover algorithms using networked quantum oracle devices. We have therefore demonstrated that universal quantum computers can access networked quantum devices in a way analogous to that by which classical computers access network resources. The method that we used to map quantum algorithms onto 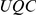 can be applied to implement and analyze other quantum algorithms.
